# Photocatalysis applications of some hybrid polymeric composites incorporating TiO_2_ nanoparticles and their combinations with SiO_2_/Fe_2_O_3_

**DOI:** 10.3762/bjnano.8.30

**Published:** 2017-01-27

**Authors:** Andreea Laura Chibac, Tinca Buruiana, Violeta Melinte, Emil C Buruiana

**Affiliations:** 1 Petru Poni Institute of Macromolecular Chemistry, 41 A Gr. Ghica Voda Alley, 700487 Iasi, Romania

**Keywords:** hybrid polymer composites, maghemite nanoparticles, photocatalysis, TiO_2_ nanoparticles, UV–visible irradiation

## Abstract

Polymer nanocomposites containing titanium oxide nanoparticles (TiO_2_ NPs) combined with other inorganic components (Si–O–Si or/and γ-Fe_2_O_3_) were prepared by the dispersion of premade NPs (nanocrystalline TiO_2_, TiO_2_/SiO_2_, TiO_2_/Fe_2_O_3_, TiO_2_/SiO_2_/Fe_2_O_3_) within a photopolymerizable urethane dimethacrylate (polytetrahydrofuran-urethane dimethacrylate, PTHF-UDMA). The physicochemical characterization of nanoparticles and hybrid polymeric composites with 10 wt % NPs (S1–S4) was realized through XRD, TEM and FTIR analyses. The mean size (10–30 nm) and the crystallinity of the NPs varied as a function of the inorganic constituent. The catalytic activity of these hybrid films was tested for the photodegradation of phenol, hydroquinone and dopamine in aqueous solution under UV or visible-light irradiation. The best results were obtained for the films with TiO_2_/Fe_2_O_3_ or TiO_2_/SiO_2_/Fe_2_O_3_ NPs. The degradation of the mentioned model pollutants varied between 71% and 100% (after 250 min of irradiation) depending on the composition of the hybrid film tested and the light applied (UV–visible light). Also, it was established that such hybrid films can be reused at least for five cycles, without losing too much of the photocatalytic efficiency (ca. 7%). These findings could have implications in the development of new nanocatalysts.

## Introduction

Over the last years, titania nanomaterials have attracted a lot of attention as they have found numerous applications in the field of dye-sensitized solar cells, Li-ion batteries, sensors, photodynamic cancer therapy or in biomaterials [1–7]. Since 1972, when Fujishima and Honda published their seminal work [8], much work has been focused on investigating the photocatalytic properties of TiO_2_ [9]. Titanium dioxide catalysts proved to be better than the other catalysts studied in literature (ZnO, SnO_2_, WO_3_, CdS) because of their superior redox ability and photoelectric properties, the long-term stability, the nontoxicity, and the low cost [10–15]. Nevertheless, the practical applications of TiO_2_ have a major drawback, namely, they are active only in UV light (<4% of sunlight) owing to their wide bandgap (3.2 eV), which absorbs photons with wavelengths shorter than 400 nm. Also, TiO_2_ nanoparticles (NPs) have a low adsorption capacity for hydrophobic molecules, and if they are used for water treatment, severe problems during separation and recycling of photocatalyst particles from aqueous suspension can appear. In order to eliminate or at least diminish the limitations mentioned, many studies were directed on obtaining TiO_2_-based materials that are active under solar/visible light. It has been demonstrated that the increase of the catalytic activity of titanium(IV) oxide in the range of visible light (λ > 430 nm) can be attained by doping the TiO_2_ network with non-metals [16–18], lanthanide ions [19–20], transitional metal ions [21–23], noble metals [24–25] or metallic oxides [26]. Other strategies can be pursued to reuse and reduce the expense caused by complex centrifugation or filtration steps of the nanostructured photocatalysts, for example, the preparation of TiO_2_ NPs with magnetic properties [27–30] or the immobilization of titania on/in diverse matrices such as glass, zeolite, ceramic, silica, graphene, and polymers [31–37]. Despite this history, few studies have been taken into account all these terms simultaneously to anticipate a feasible variant. Consequently, the challenge remains for the whole research community to address these crucial aspects including the maximization of the photocatalytic efficiency.

An interesting route to reach this goal is the use of iron(III) added to titanium dioxide photocatalysts, which improves the photocatalytic activity under visible light reducing the recombination rates of the photo-excited carriers [38]. Also, the immobilization of TiO_2_ photocatalysts in a polymer matrix allowing a re-use seems to be beneficial in contrast to the colloidal photocatalytic systems. In fact, the fabrication of such composites from conjugated organic polymers (polypyrrole, polyaniline) and TiO_2_ NPs [39–41] or other polymer matrices as polymethylmethacrylate, polyurethanes, polyvinylidene fluoride, polyethersulfone, cellulose acetate, and polyvinyl alcohol [42–43], together with their testing in water treatment were reported in literature.

The aim of this study was to develop new hybrid polymeric materials with four types of titania nanoparticles, namely: nanocrystalline TiO_2_, TiO_2_ with Si–O–Si sequences (TiO_2_/SiO_2_), TiO_2_ with maghemite nanoparticles (TiO_2_/Fe_2_O_3_), and TiO_2_ with Si–O–Si and maghemite (TiO_2_/SiO_2_/Fe_2_O_3_). For the preparation of these composites premade nanoparticles were dispersed into urethane dimethacrylate followed by photopolymerization of the monomer to form a highly cross-linked film. The choice to use the polyacrylic matrix can be explained by the excellent transparency, chemical and weather resistance, mechanical stability, adhesion, and film forming properties [44]. Besides, the photopolymerization reactions are induced through UV irradiation and can be conducted in very short time (seconds/minutes) at room temperature with no degradation of sensitive molecules [45–46]. The impact of the addition of different inorganic parts to the TiO_2_ NPs on the nanocomposite properties, including their photocatalytic performance in degrading hydroxybenzene derivatives under UV and visible light, was investigated using hybrid films in aqueous solutions. The motivation for this selection is that the phenolic compounds play a major role among the various organic pollutants that cause environmental hazard, being a part of the widely used drugs, pesticides, dyes, and plastics. On the basis of this information, we tried to prepare stable photocatalytic materials with good activity against phenolic pollutants under UV–visible light starting from TiO_2_ NPs with and without Si–O–Si sequences and/or Fe_2_O_3_ NPs immobilized in a polymer network suitable for several cycles of utilization. It is thought that the use of such photopolymerized films in water treatment applications could be advantageous because the work with NPs powders, which require separation techniques for their re-use, can be avoided.

## Results and Discussion

### Synthesis and physicochemical characterization of nanoparticles

**Figure 1:**
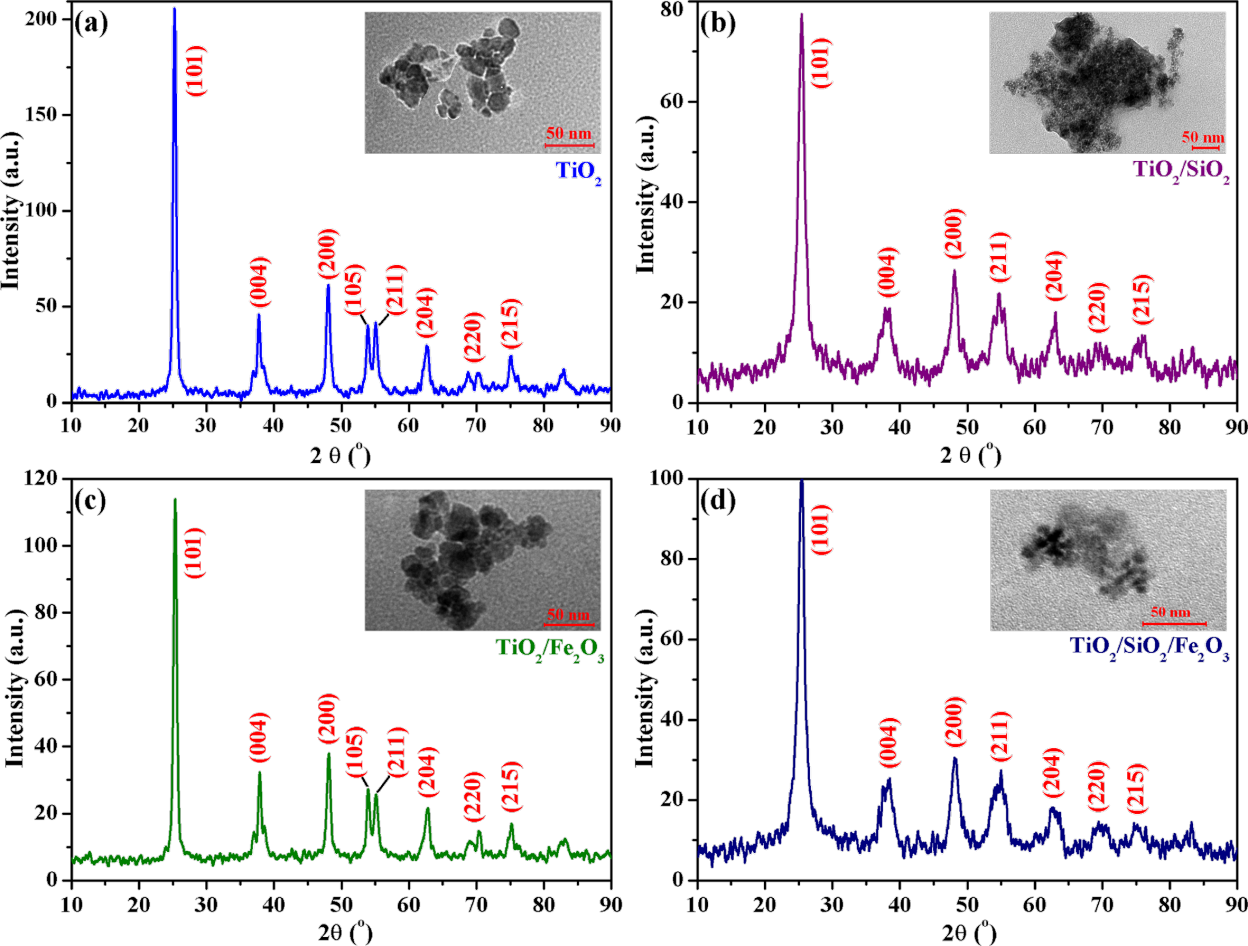
XRD patterns and TEM images (inset) for (a) TiO_2_, (b) TiO_2_/SiO_2_, (c) TiO_2_/Fe_2_O_3_ and (d) TiO_2_/SiO_2_/Fe_2_O_3_ nanopowders.

Titania nanoparticles (TiO_2_) and titania nanoparticles mixed with Si–O–Si (TiO_2_/SiO_2_), γ-Fe_2_O_3_ (TiO_2_/Fe_2_O_3_) or Si–O–Si and γ-Fe_2_O_3_ (TiO_2_/SiO_2_/Fe_2_O_3_) were obtained by the sol–gel method, using titanium isopropoxide as precursor. The crystalline structures of the resulting inorganic materials were firstly identified by XRD. The XRD spectra of TiO_2_ (a), TiO_2_/SiO_2_ (b), TiO_2_/Fe_2_O_3_ (c) and TiO_2_/SiO_2_/Fe_2_O_3_ (d) are given in Figure 1.

The X-ray diffraction pattern of TiO_2_ particles shows peaks at 2θ = 25.3°, 37.8°, 48.0°, 54.0°, 55.1° 62.8°, and 75.0°, assigned to the (101), (004), (200), (105), (211), (204), and (215) planes, respectively, confirming the formation of nanocrystalline anatase. This characteristic is essential from the point of view of application since the crystal structure of TiO_2_ significantly affects the photocatalytic activity [47]. The average crystallite size of anatase, calculated by applying the Scherrer equation to the major diffraction peak was found to be 21.8 nm. Besides, the TEM image of TiO_2_ nanoparticles (Figure 1a, inset) sustains that spherical TiO_2_ NPs with a relatively uniform size distribution (particle dimensions in the range of 25–30 nm) were obtained.

In the second type of nanoparticles (TiO_2_/SiO_2_ – Figure 1b), the two inorganic components interact through hydrolysis and condensation reactions, leading finally to clusters or small associations of titania domains linked by Si–O–Si sequences. The XRD pattern reveals the effect of the other inorganic components on the crystalline phase of TiO_2_. The presence of amorphous Si–O–Si linkages in titania nanoparticles lead to a decrease in the intensity and a broadening of the peak corresponding to the (101) plane of anatase. This finding suggests the formation of smaller anatase crystallites in the case of TiO_2_/SiO_2_ NPs due to a limitation effect exerted by the presence of the SiO_2_ phase in the growth of TiO_2_ grains [48]. However, the crystalline structure of TiO_2_ nanoparticles was not significantly modified by the SiO_2_ domains. The estimated size of the anatase crystallites according to the Scherrer equation is about 12.8 nm. The TEM image evidenced the association of small nanoparticles (10–20 nm) with Si–O–Si linkages to form larger structures of about 150–250 nm.

The XRD pattern of the mixture TiO_2_ NPs/maghemite (Figure 1c) contains mainly the diffraction peaks specific to anatase TiO_2_. The intensity of the peaks corresponding to 1 wt % γ-Fe_2_O_3_ was too small in comparison to that of anatase. Still, the shape of the pattern indicated a good crystallinity and the average TiO_2_ crystallite size determined from the main (101) anatase peak was 20.2 nm. The TEM image (Figure 1c, inset), sustains the formation of TiO_2_ NPs where some small maghemite particles (about 2 to 5 nm) can be seen.

The XRD pattern for the TiO_2_ nanoparticles linked through Si–O–Si sequences and combined with maghemite nanoparticles (Figure 1d) displays the characteristic reflection peaks for anatase, even if these peaks are broader and less intense than that measured for anatase nanopowder. In this case, the average crystallite size of anatase calculated according to the Scherrer equation was 13.6 nm, confirming the effect of the SiO_2_ phase on the growth of anatase grains, as previously observed for TiO_2_/SiO_2_ NPs. Moreover, the same association tendency as that of TiO_2_/SiO_2_ NPs can be seen in the TEM image. The formed aggregates are smaller (50–100 nm) and the nanoparticles are more clearly visible.

**Figure 2:**
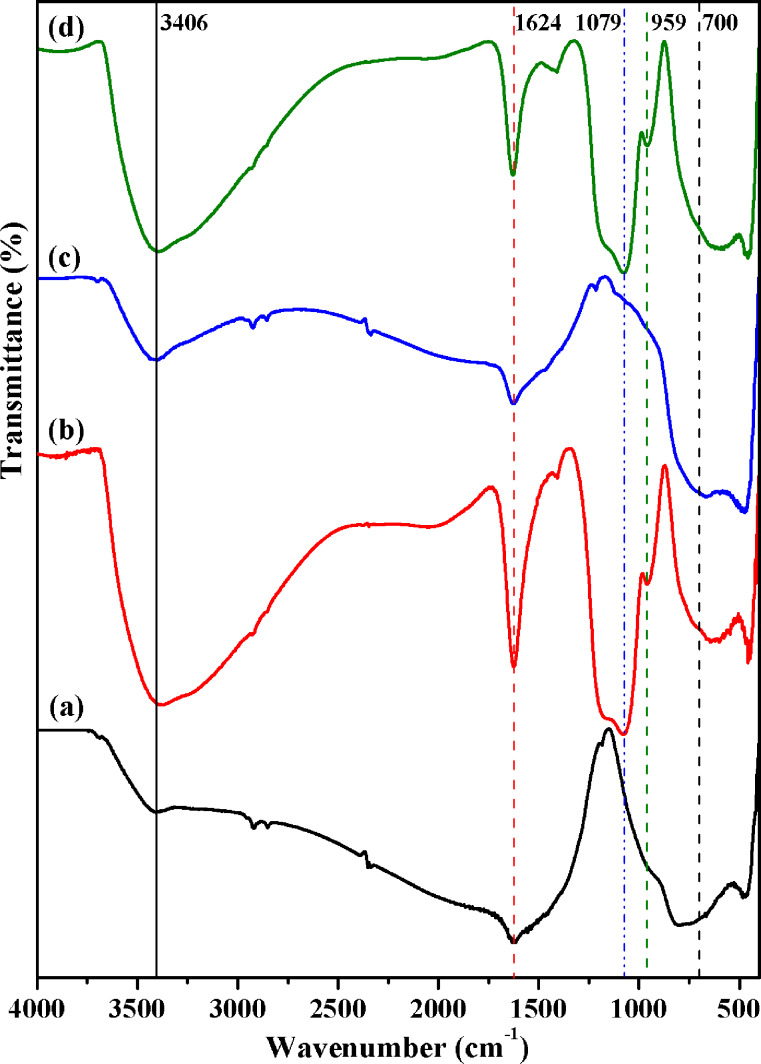
FTIR spectra of TiO_2_ (a), TiO_2_/SiO_2_ (b) TiO_2_/Fe_2_O_3_ (c) and TiO_2_/SiO_2_/Fe_2_O_3_ (d).

Figure 2a shows the FTIR spectrum of pure TiO_2_ NPs. There is a broad characteristic absorption band at 400–700 cm^−1^ attributable to the Ti–O–Ti network structure, while the bands at 3406 and 1624 cm^−1^ are from water [49] that was adsorbed because of the hydrophilic nature of the nanoparticles. Further, the Si–O–Si domains in TiO_2_/SiO_2_ NPs (Figure 2b) generated the strong absorption bands of the stretching vibration of Si–O–Si bonds at about 1079 cm^−1^, suggesting the formation of silica layers. The OH groups (Si–OH and Ti–OH) led to a broad absorption band at 3400 cm^−1^, while the absorption band located at 959 cm^−1^ is due to the vibration of Ti–O–Si bonds [50–51]. The FTIR spectrum for TiO_2_/Fe_2_O_3_ NPs (Figure 2c) is quite similar to that of pristine TiO_2_ NPs, and the maghemite Fe–O absorption bands at about 570 cm^−1^ are overlapped by those of titanium dioxide. Likewise, in the FTIR spectrum of TiO_2_/SiO_2_/Fe_2_O_3_ nanopowder (Figure 2d) the absorption bands are analogous to those already discussed. Furthermore, in all FTIR spectra two weak absorption bands at 2800–2900 cm^−1^ can be distinguished , which may indicate the existence of residual hydrocarbon specious derived from the alkoxy groups (such as from TiOR or SiOR) [51].

### Synthesis and characterization of hybrid composites

**Figure 3:**
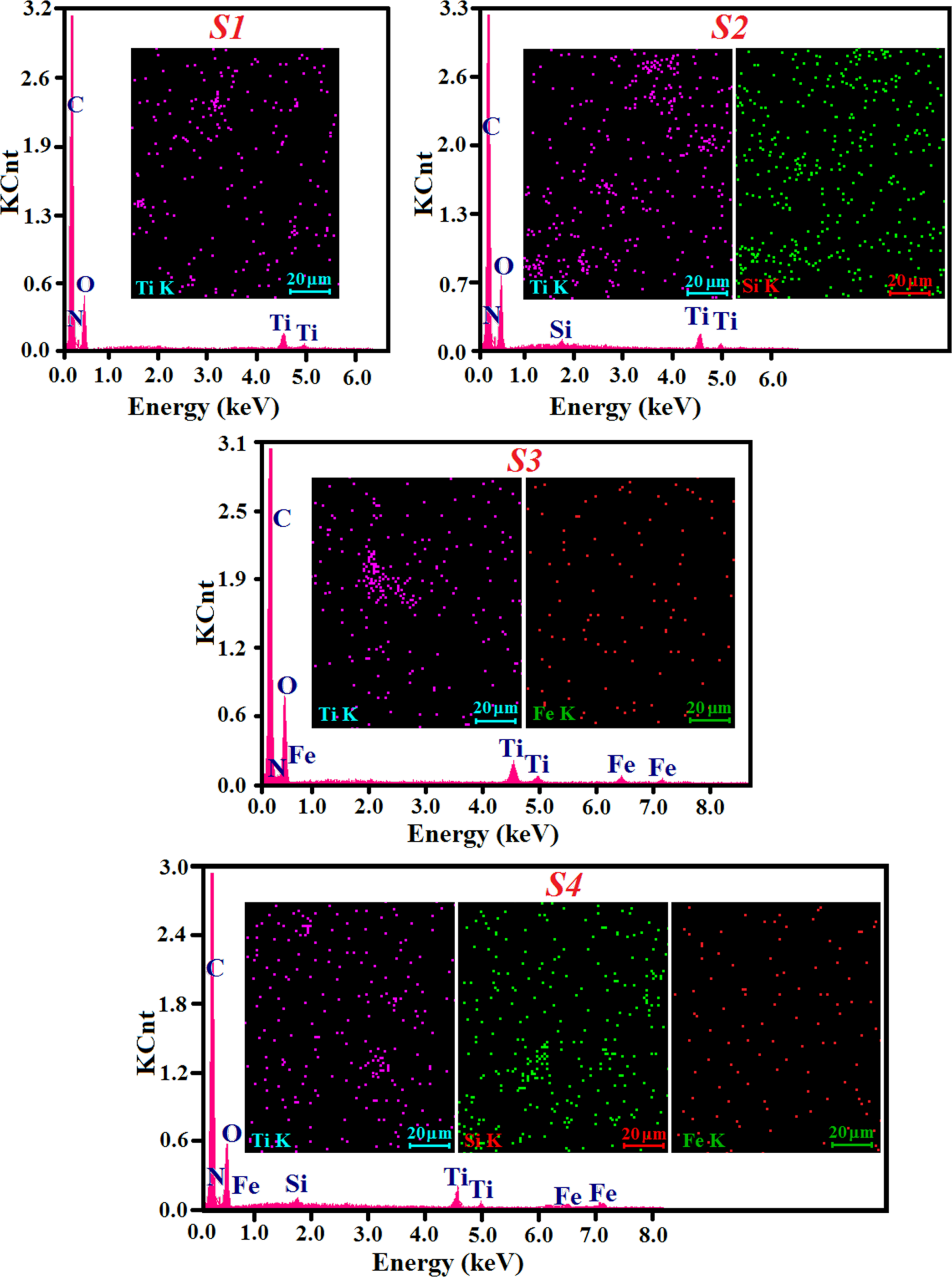
EDX spectra of S1–S4 hybrid composites recorded in cross section and mappings of titanium, silicon and iron.

As mentioned, the dispersion of inorganic nanoparticles into suitable organic matrices prevents their aggregation and furnishes a convenient handling of the NPs, besides reducing toxicity and enhancing the chemical stability [52]. Hence, 10 wt % TiO_2_ NPs (pure or in different combinations) were incorporated into a viscous urethane dimethacrylate (PTHF-UDMA) to be UV photopolymerized in the presence of Irgacure 819 as photoinitiator. This process led to the formation of flexible polymer networks (S1–S4). Energy-dispersive X-ray spectroscopy (EDX) was employed to evaluate the chemical composition (qualitative and quantitative) of all hybrid nanocomposites containing TiO_2_ NPs and also to investigate the spatial distribution of the elements through the X-ray elemental mapping images. In the EDX patterns (Figure 3), the Ti, Si and Fe signals confirmed the existence of inorganic nanoparticles, while the organic matrix is represented by the characteristic C, O and N peaks, supporting thus the formation of hybrid materials. In addition, the elemental mapping images for titanium, silicon and iron atoms, registered on the SEM images of cross-section of the composite films suggested a relatively uniform distribution of the inorganic components within the polymer matrix.

**Figure 4:**
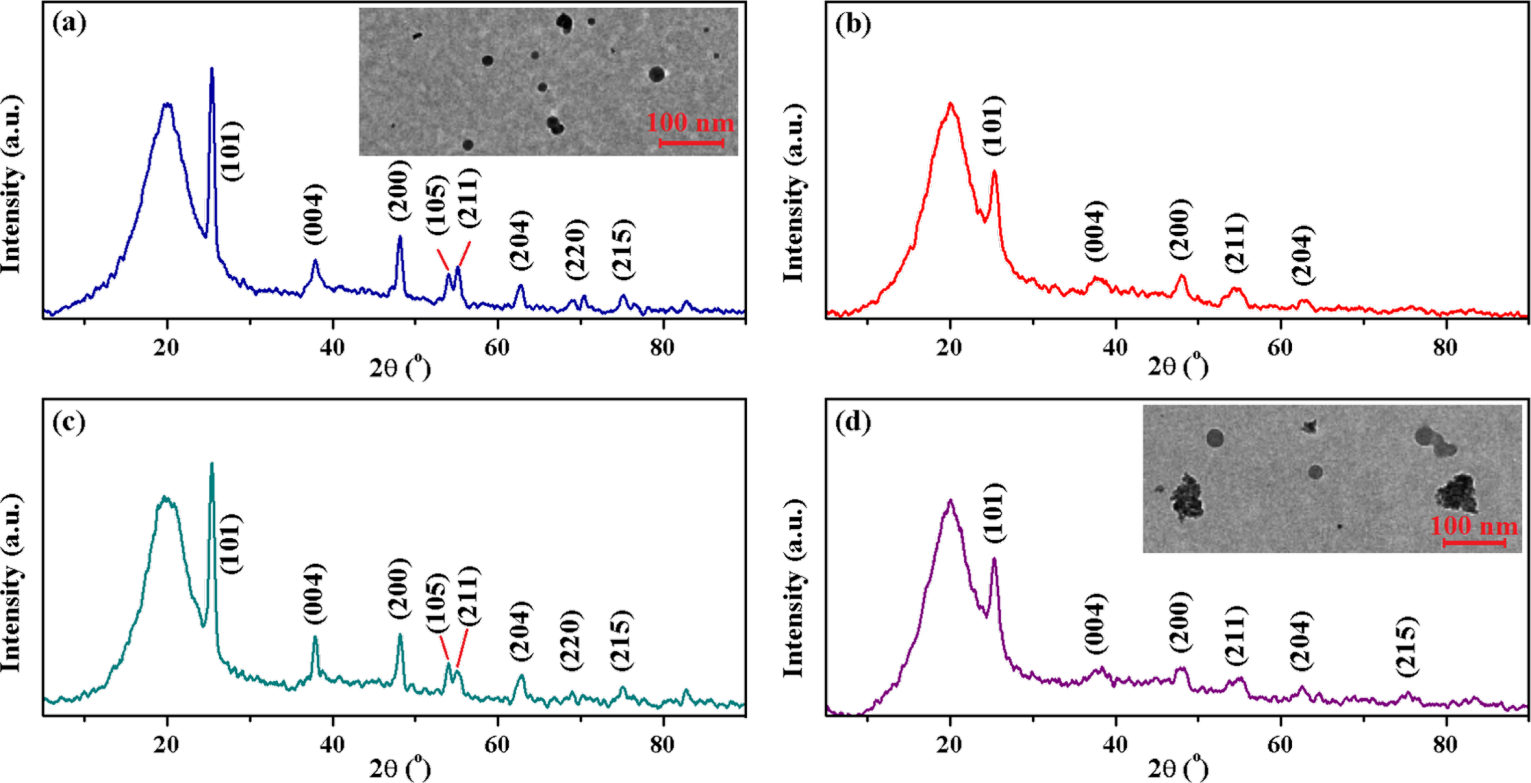
XRD patterns and TEM images (inset) for (a) S1, (b) S2, (c) S3 and (d) S4 hybrids.

The hybrid polymeric nanocomposites with TiO_2_ were also characterized by X-ray diffraction. The collected patterns are plotted in Figure 4. Although the presence of the amorphous organic matrix weakens the diffraction peaks, the appearance of anatase TiO_2_ peaks can be detected. Moreover, a broad peak at around 19.5° corresponding to the amorphous structure of the polymer matrix can be identified in all XRD patterns. The TEM images revealed that the premade TiO_2_ particles (pure or mixed with Si–O–Si or/and Fe_2_O_3_) are highly stable and uniformly dispersed in the polymeric matrix, as can be observed for the hybrid composites S1 and S4 in the inset of Figure 4a and Figure 4d, respectively. Therefore, it is clear that the introduction of the TiO_2_ nanoparticles in a polymer template avoids their agglomeration.

**Figure 5:**
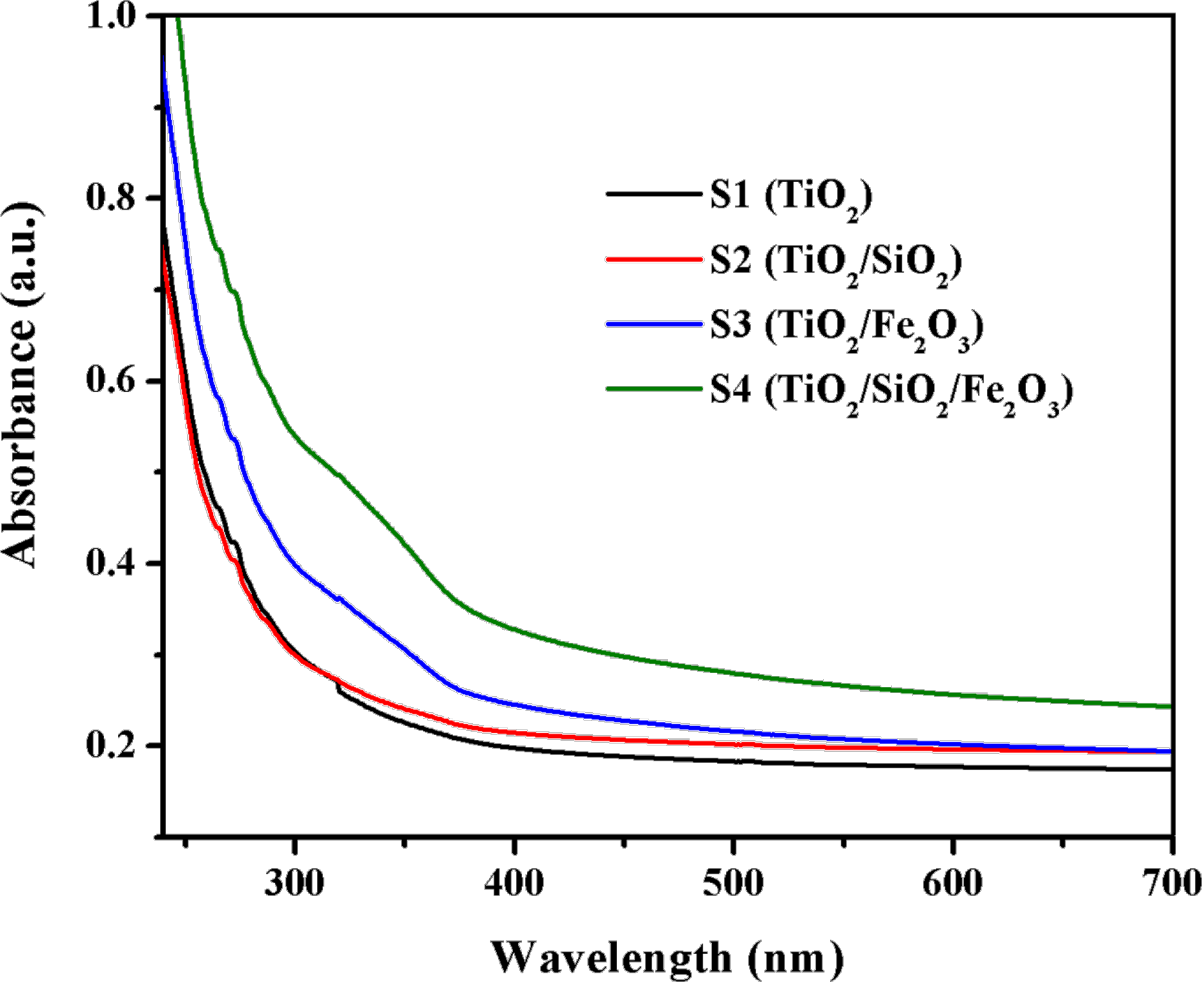
UV–vis absorption spectra of hybrid polymeric films S1–S4.

The hybrid polymeric films, S1–S4 (thickness of about 200 μm), with hybridized TiO_2_ NPs were further examined through UV–vis analysis and the absorption spectra are given in Figure 5.

It should be pointed out that the samples S1 (with TiO_2_ NPs) and S2 (with TiO_2_/SiO_2_ NPs) films have no absorption in the visible region. These films absorb only UV radiation. The combination of titania with maghemite nanoparticles induced an increase of the absorption for S3 and S4 composites above 300 nm, a convenient characteristic for photocatalytic applications.

### Evaluation of photocatalytic activity of the hybrid composites

The photocatalytic performance of the hybrid composites incorporating TiO_2_ NPs alone or together with other inorganic components (Si–O–Si sequences, γ-Fe_2_O_3_ NPs) was investigated following the degradation of three hydroxybenzenes, namely with one –OH group (phenol), with two –OH groups (hydroquinone), and with two –OH groups and an amine function (dopamine). The photodegradation experiments for these model pollutants were performed under ambient conditions, under UV irradiation with low intensity (ca. 8 mW/cm^2^) to imitate the UV radiation from solar light, as well as under irradiation with visible light. The degradation process was measured by the intensity changes of the absorption band of the hydroxybenzenes as a function of the irradiation time. To confirm the photocatalytic activity of the hybrid composites with TiO_2_ NPs, three control experiments (not shown) were carried out. First, the pollutant solutions were kept in dark in the presence of the investigated films (S1–S4); second, they were illuminated (UV–visible radiation) in the absence of the polymer composites, and third, the investigated solutions were irradiated in the presence of the pure polymer film (without TiO_2_ photocatalysts). In all cases, the pollutant solutions showed only slight degradation. Therefore, an efficient degradation of toxic organic pollutants needs the presence of light and TiO_2_-based catalysts. In general, the photocatalytic mechanism of the materials incorporating TiO_2_ NPs can be described as follows: The TiO_2_ nanoparticles absorb the UV–visible radiation and charge carriers (holes and electrons) are photogenerated [12,25,53]. The electrons migrate inside the conduction band, and thus appear new holes that are filled by other electrons nearby. The process is resumed until the electrons and holes reach the surface. At this point, the degradation of organic compounds into CO_2_ and H_2_O begins. It is caused by reactive radicals (•OH, •O_2_^−^) produced through the interactions between the charge carriers from the surface with the adsorbed molecules (e.g., water and molecular oxygen) [25,54].

**Figure 6:**
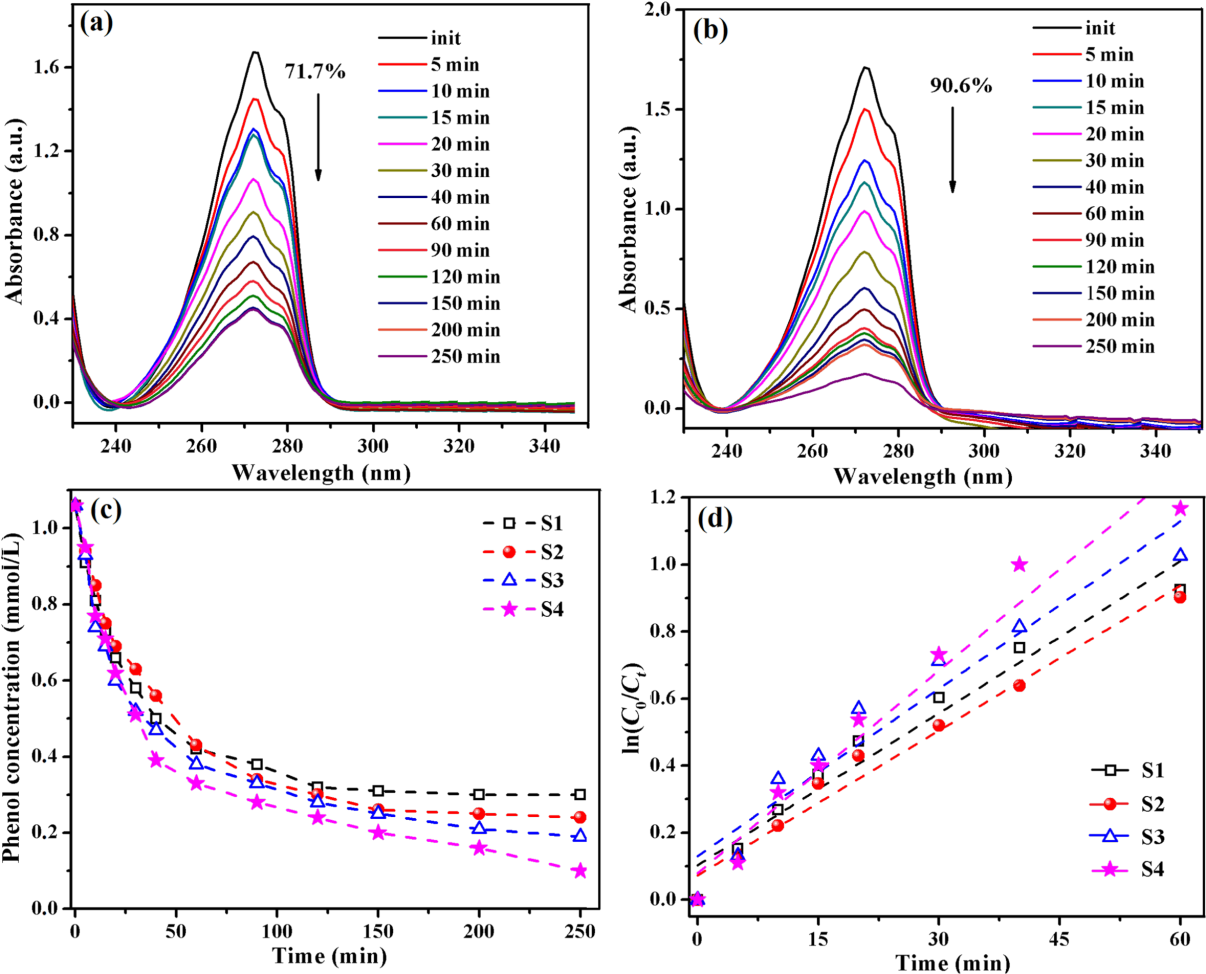
Changes of UV–vis absorption spectra of an aqueous phenol solution in the presence of S1 (a) and S4 (b) films used as catalysts monitored as a function of UV irradiation time; temporal evolution of the phenol concentration (c) and fitted curves for the kinetic estimation of phenol photodegradation (d) in presence of S1–S4 films.

For this study, the photodecomposition process of an aqueous solution of phenol (1.06 × 10^−3^ M) in the presence of hybrid materials with TiO_2_ nanoparticles, pure or combined with SiO_2_ and/or Fe_2_O_3_, was investigated by monitoring the characteristic absorption band of phenol at λ = 270 nm. Figure 6a,b shows the UV–vis absorption spectra collected during UV irradiation in the presence of S1 and S4 hybrid films. As can be observed, the absorption band of phenol underwent a gradual decrease because of its photodegradation without any change in the shape and position (after 250 min irradiation). Furthermore, for determining the phenol concentration as a function of irradiation time we used the Lambert–Beer law (*A* = ε·*l*·*c*), and the resulting plots are given in Figure 6c. It was found that the phenol degradation efficiency depends on the hybrid film employed as catalyst, and the photocatalytic performance is improved by the addition of other inorganic substances in the photopolymerizable macromer. The degrees of phenol photolysis attained after 250 min of UV irradiation in the presence of our hybrid composites are as follows: 71% for S1 (with TiO_2_), 77% for S2 (with TiO_2_/SiO_2_), 82% for S3 (with TiO_2_/Fe_2_O_3_), and 90% for S4 (with TiO_2_/SiO_2_/Fe_2_O_3_).

The improved photocatalytic activity of the polymeric films containing TiO_2_ hybridized with various elements could be explained through the synergic contributions of TiO_2_, the Si–O–Si phase or/and maghemite nanoparticles. It can be assumed that the polymeric films with hybridized TiO_2_ have an excellent ability to adsorb aromatic compounds through π–π stacking and other molecular interactions, enhancing the catalysis effect due to the presence of other inorganic components. Also, in the case of the composites with TiO_2_/Si–O–Si (S2) or TiO_2_/Fe_2_O_3_/Si–O–Si (S4) the presence of a higher number of hydroxyl groups favoured the photocatalytic activity due to a greater capacity for the absorption of oxygen [55–57].

Besides, the rate constants (*k*) for the phenol degradation process under UV irradiation in the presence of the hybrid films were determined according to equation:


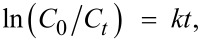


where *C*_0_ and *C_t_* are the values of the concentration at times *t*_0_ and *t*, respectively, and *k* is the rate constant. Along the interval from 0 to 60 min, the experimental data of the photodegradation reactions exhibit first-order kinetics, as can be noticed from Figure 6d. The apparent rate constants for the reactions were calculated from the slope of the straight fitting line, and the found values were *k* = 15.13 × 10^−3^ min^−1^ (S1), 14.42 × 10^−3^ min^−1^ (S2), 16.65 × 10^−3^ min^−1^ (S3) and of 20.17 × 10^−3^ min^−1^ (S4).

**Figure 7:**
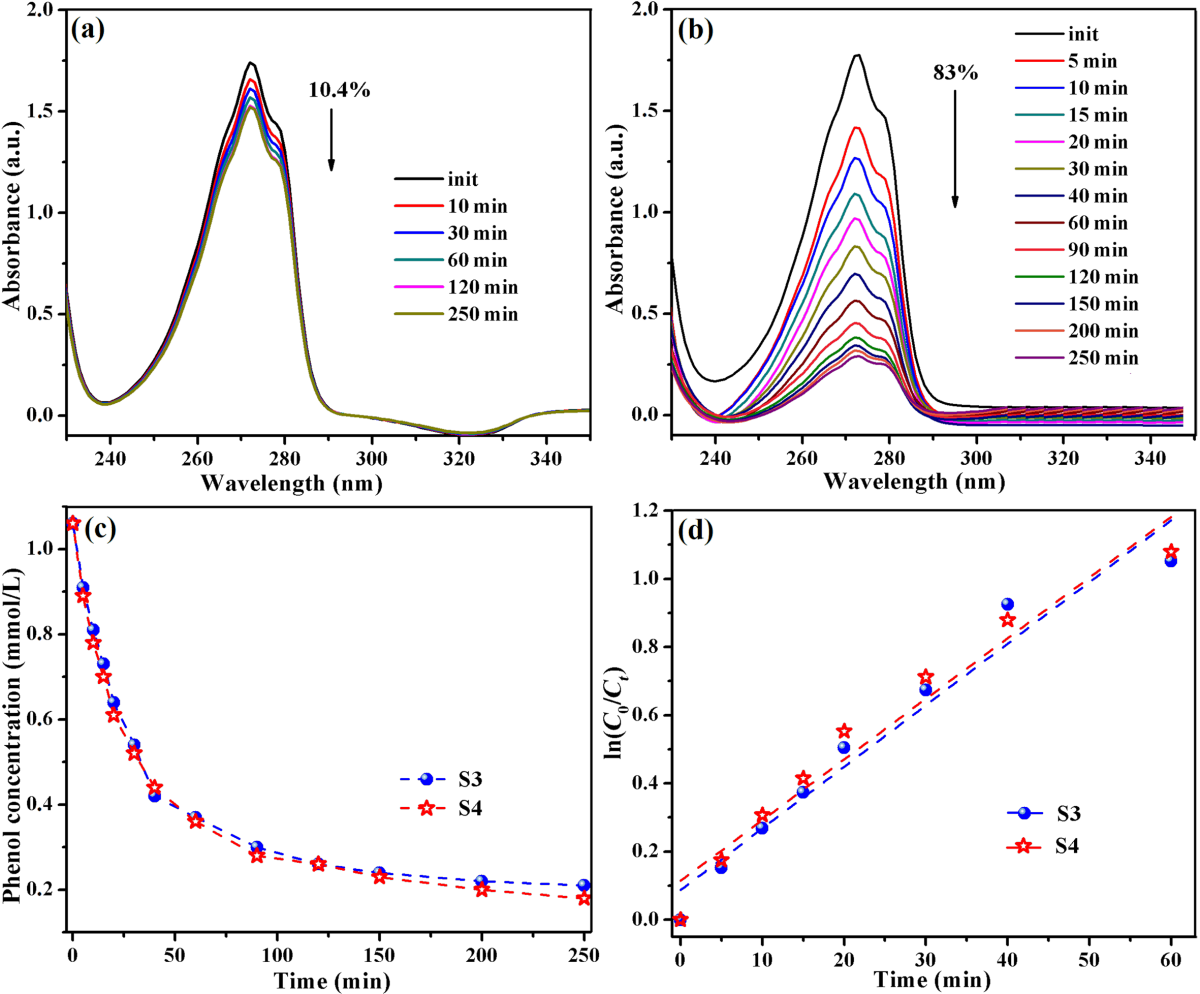
Changes of UV–vis absorption spectra of an aqueous phenol solution in the presence of S1 (a) and S4 (b) hybrids monitored as a function of the time of visible-light irradiation; temporal evolution of the phenol concentration (c) and fitted curves for the kinetic estimation of phenol photodegradation (d).

Analogous experiments for the photodecomposition of phenol in the presence of hybrid films under visible light were performed. As expected, S1 exhibited a very low photodegradation activity (Figure 7a), only 10% of phenol being reduced after 250 min of visible-light irradiation. The UV–vis absorption spectra measured in the presence of S4 hybrid (Figure 7b) show that phenol is decomposed with increasing irradiation time. The phenol photolysis degree under visible light in the presence of S3 and S4 films (with TiO_2_ and Fe_2_O_3_ NPs) is about 80% and 83%, respectively (Figure 7c). This means that the photodecomposition of phenol is similarly effective as under UV illumination if a proper catalyst is used. The apparent rate constants were around 18 × 10^−3^ min^−1^ (Figure 7d) for the two photocatalysts S3 and S4.

The mineralization of phenol, i.e., the complete degradation of phenol and its decomposition intermediates to CO_2_, was investigated by measuring the concentration of total organic carbon (TOC) in the solutions irradiated with visible light for 250 min in presence of the S4 polymeric film. The TOC analysis indicated a phenol mineralization of about 46%, the result suggesting that the speed of TOC removal is slower (approximately half) than the phenol degradation into intermediate species.

**Figure 8:**
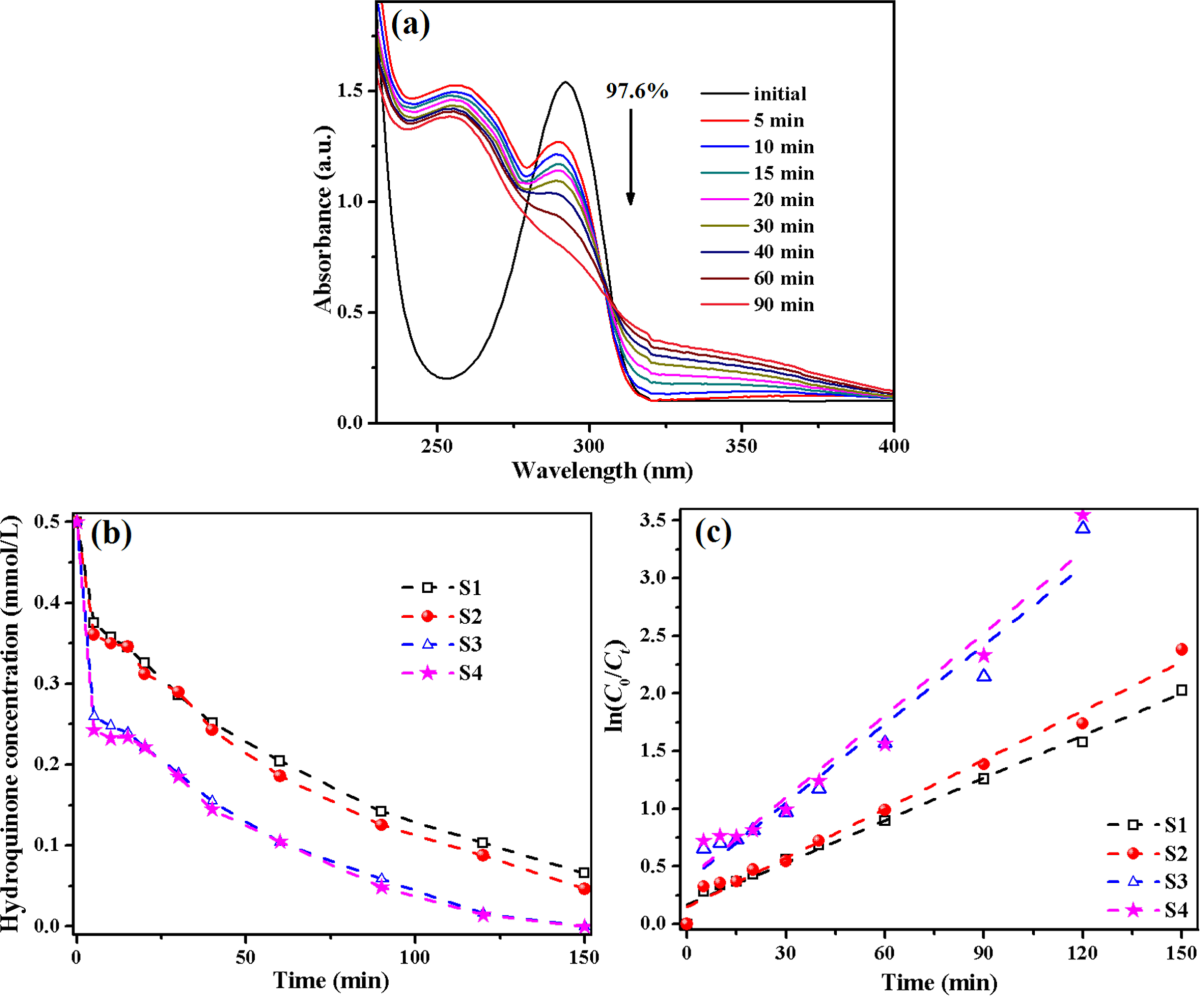
UV–vis absorption spectra of an aqueous hydroquinone solution in the presence of S4 film as a function of the UV irradiation time (a); temporal evolution of the hydroquinone concentration (b) and fitted curves for the kinetic estimation of hydroquinone photodegradation (c) in presence of S1–S4 hybrids.

A different photobehaviour was observed for aqueous hydroquinone solutions (0.5 × 10^−3^ M) in the presence of our hybrids catalysts S1–S4. Figure 8a displays the hydroquinone UV–vis absorption spectra under UV irradiation in the presence of S4 film. It can be observed that after only 5 min of UV irradiation the typical absorption band of hydroquinone (λ = 292 nm) is reduced by 51%, and in the spectrum appears a new well-defined band at 260 nm, characteristic to *p*-benzoquinone. This finding suggests that 51% of hydroquinone is oxidized to *p*-benzoquinone in the first 5 min of UV illumination, and the two compounds (hydroquinone and *p*-benzoquinone) are simultaneously decomposed with increasing irradiation time. After 90 min of UV irradiation more than 97% of hydroquinone is removed from the system or converted to *p*-benzoquinone, while *p*-benzoquinone needs a longer irradiation time for a complete elimination. For instance, just 60% of the derivative was reduced after the same period of time. As can be deduced from the temporal evolution of the hydroquinone concentration plots (Figure 8b) the catalysts containing maghemite and TiO_2_ NPs (S3 and S4) have a better efficiency than the ones with only titania (S1 and S2 hybrids). It is obvious that after 150 min of UV irradiation the hydroquinone was totally decomposed by the catalysts S3 and S4.

**Figure 9:**
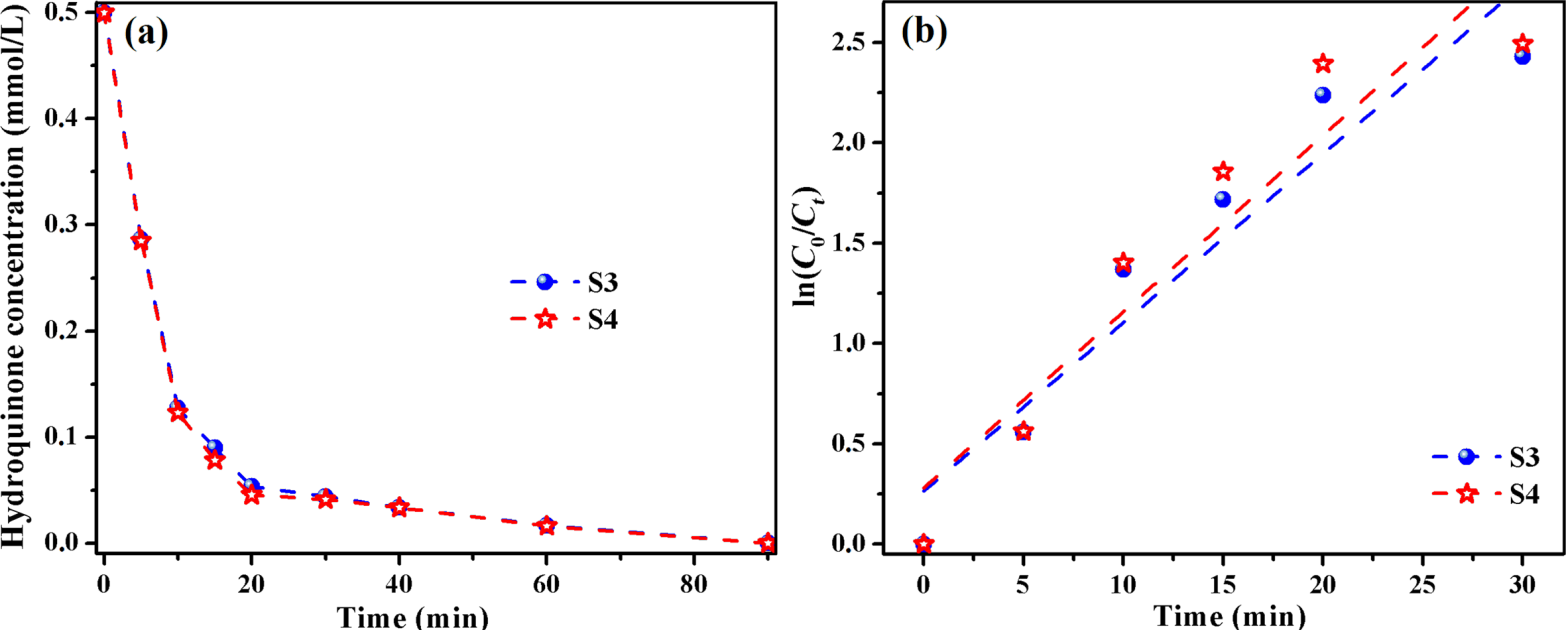
Temporal evolution of the hydroquinone concentration (a) and fitted curves for the kinetic estimation of hydroquinone photodegradation (b) under visible light in presence of S3 and S4 films.

The hydroquinone photolysis is a process with first-order kinetics and the apparent rate constants (*k*) were found to be 12.24 × 10^−3^ min^-1^ (S1), 14.18 × 10^−3^ min^−1^ (S2), 22.81 × 10^−3^ min^−1^ (S3) and 23.75 × 10^−3^ min^−1^ (S4). The higher catalytic efficiency of S3 and S4 can be related to the presence of TiO_2_ NPs and maghemite nanoparticles. According to the literature [38], the electronic configuration of Fe^3+^ with half-filled orbitals induces the narrowing of the energy gap and the attenuation of the recombination of electrons and holes by capturing photogenerated carriers. Also, the Fe^3+^ cation can separate the photo-excited electrons and holes and can extend their lifetime by acting as a temporary trapping site for photo-induced electrons and as a shallow capturing site for photo-induced holes [27,38]. Therefore, the hydroquinone photodecomposition in visible light takes place only in the presence of S3 and S4 catalysts incorporating the TiO_2_/Fe_2_O_3_ and TiO_2_/SiO_2_/Fe_2_O_3_, respectively. The two catalysts have almost the same activity (Figure 9a), and after 90 min of visible-light irradiation the hydroquinone is completely removed from the aqueous solutions. The lack of photocatalytic activity of S1 (with TiO_2_) and S2 (TiO_2_/SiO_2_) hybrid films under visible light may be related to the absorption of TiO_2_ NPs in the UV region. On the other hand, the presence of Fe^3+^ cation extends the UV–vis absorption of TiO_2_ into the visible domain [38], as can be seen in the UV–vis spectra of the hybrid films. The apparent rate constants for hydroquinone photodegradation process under visible-light irradiation, which follows first-order kinetics, are around 8.5 × 10^−2^ min^−1^ (Figure 9b) for the two photocatalysts S3 and S4. We must underline that the hydroquinone degradation under UV or visible light occurs faster than phenol photolysis due to the presence of two –OH groups, which yield a quicker access to catalyst. In the same time, the secondary product appearing in the system (*p*-benzoquinone) is almost eliminated after 250 min of irradiation.

**Figure 10:**
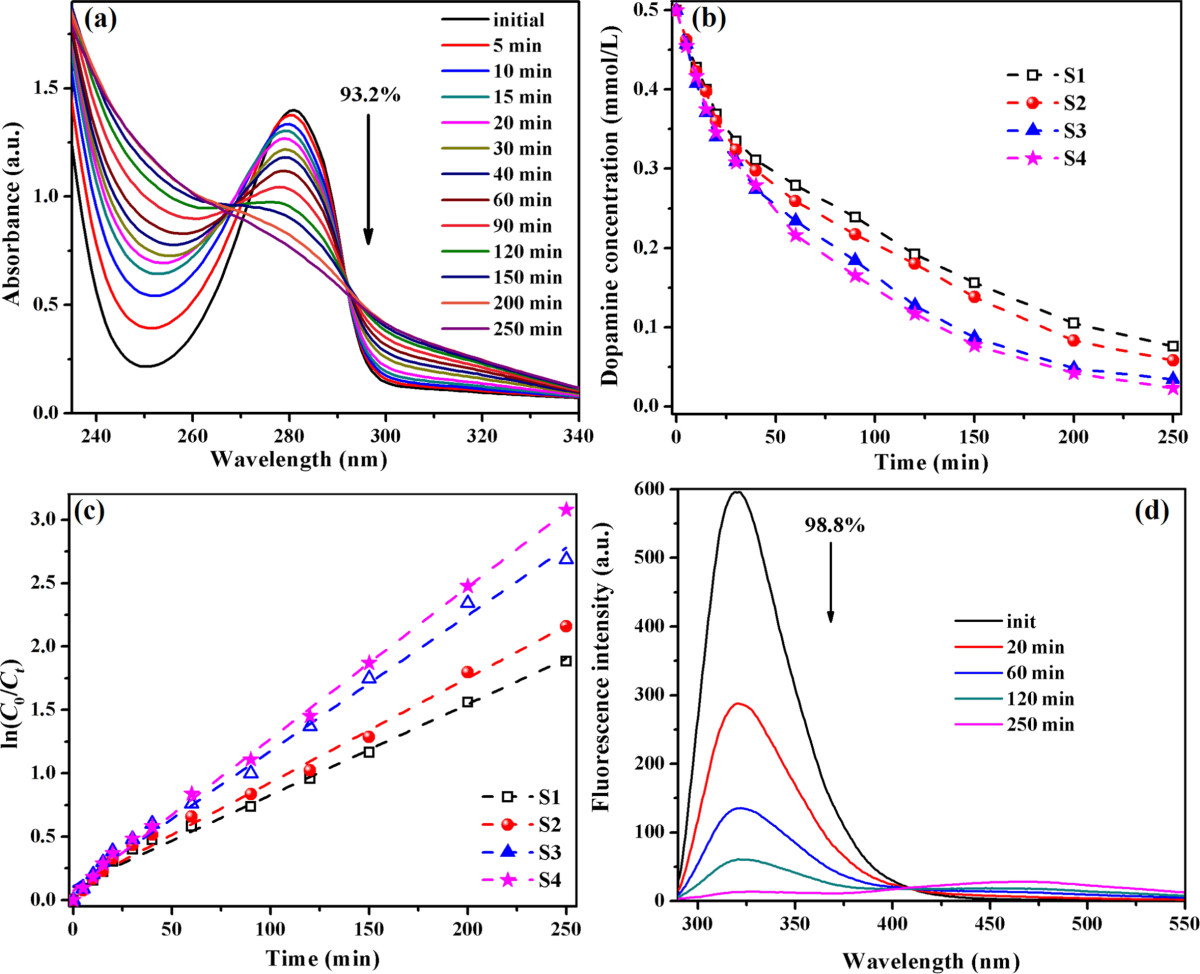
Changes of UV–vis absorption spectra of an aqueous dopamine solution in the presence of S3 as a function of the UV irradiation time (a); temporal evolution of the dopamine concentration (b) and fitted curves for the kinetic estimation of dopamine photodegradation (c) in presence of composites S1–S4; fluorescence spectra of aqueous dopamine solution in the presence of S4 as a function of the UV irradiation time (d).

We also tested the performance of our catalysts in the decomposition of dopamine, which has a catechol structure (a benzene ring with two hydroxyl side groups) and an amine group attached via an ethyl chain. From the best of our knowledge, the dopamine photodegradation with TiO_2_-based catalysts is reported first time. The photodecomposition process of an aqueous solution of dopamine (0.5 × 10^−3^ M) in the presence of hybrid films S1–S4 was evaluated by monitoring the absorption band positioned at λ = 280 nm, which is reduced during UV irradiation, as is shown in Figure 10a for the S3 catalyst. By determining the dopamine concentration as a function of the irradiation time (Figure 10b) it is clear that better results were obtained for the composites including Fe_2_O_3_ NPs (S3 and S4). Thus, UV irradiation of the S1 film yielded a dopamine photolysis degree of 85%, S2 yielded 88% dopamine, S3 yielded 93% after 250 min, and 95% of dopamine was decomposed with the S4 film. The photolysis of dopamine under UV light follows first-order kinetics and the rate constants ranged between 7.17 × 10^−3^ and 11.97 × 10^−3^ min^−1^ (Figure 10c). The occurrence of an isosbestic point in the UV–vis spectra may lead to the idea that the dopamine decomposition takes place in a single step: dopamine is oxidized very fast to the *o*-quinone derivative of dopamine, and then TiO_2_ NPs could induce the breaking of the linkage between the two ketonic functions. This hypothesis is supported by the fluorescence behaviour of the dopamine aqueous solution under UV irradiation in the presence of the S4 film (Figure 10d). The characteristic emission band of dopamine (λ = 320 nm) is reduced by 98.8% after 250 min. At the same time, the possibility of cyclization with the formation of aminochrome cannot be excluded. However, a complete elucidation of the dopamine degradation will be the subject of a future work.

**Figure 11:**
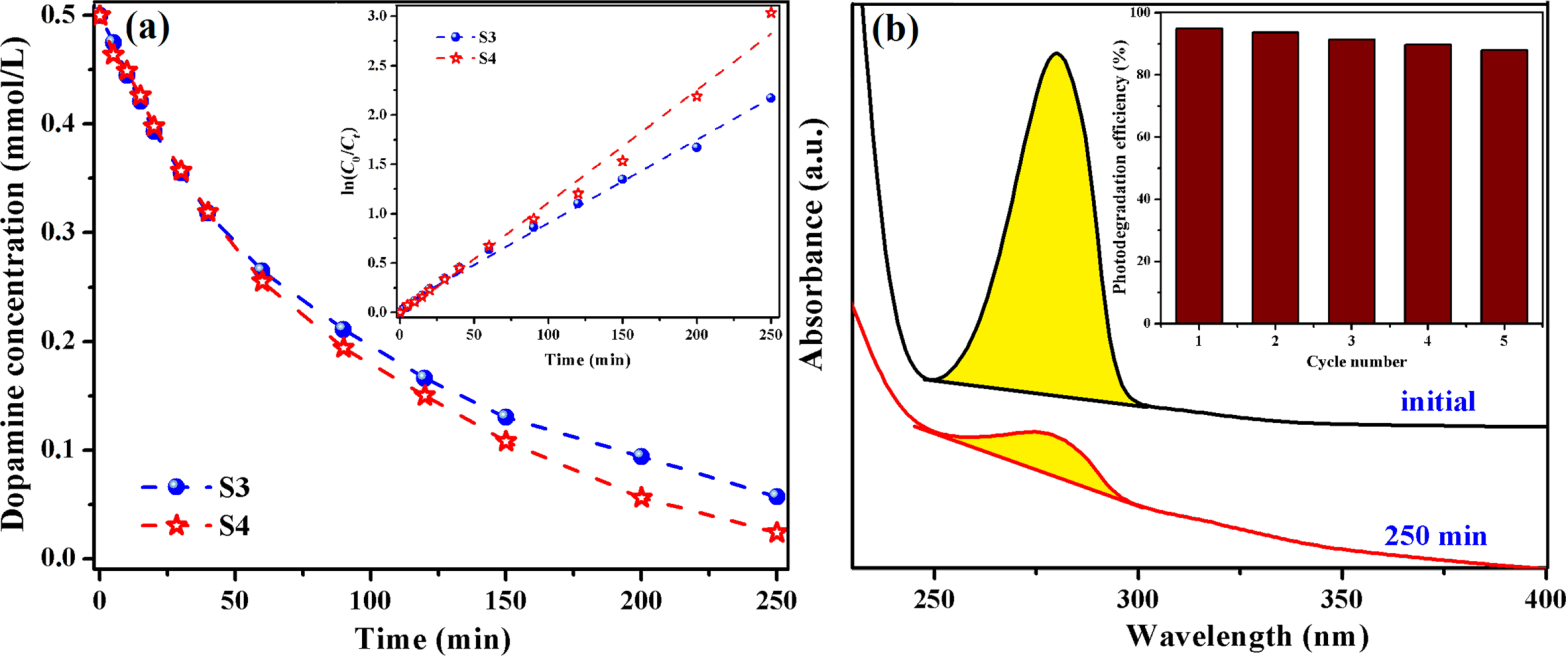
Dopamine concentration and fitted curves for the kinetic estimation (inset) of dopamine photodegradation under visible light in the presence of S3 and S4 hybrid composites (a); changes of the UV–vis absorption spectra of an aqueous dopamine solution in presence of S4 composite film under visible irradiation (250 min) after five cycles of utilization (inset: effect of the re-use of S4 catalyst on the photodegradation efficiency of dopamine in the aqueous solution under visible light) (b).

Following then the dopamine degradation under visible-light irradiation, it was observed that the films S3 and S4 lead to a decomposition, namely to 89% and 95%, respectively (Figure 11a). In the first 30 min of irradiation both catalysts have a similar activity but after 250 min of irradiation, the rate constants were 8.4 × 10^−3^ min^−1^ (S3) and 11.4 × 10^−3^ min^−1^ (S4).

In order to demonstrate the stability and the reusability of the prepared polymeric photocatalysts, the S4 film used in the dopamine decomposition under visible-light irradiation was removed from the reaction solution and reused in the next cycles. As shown in Figure 11b, in the fifth cycle of use, the S4 composite film yielded a photolysis degree of 88% of dopamine and the photocatalytic efficiency remained quite stable, decreasing by only 7%. So, it was demonstrated that the hybrid polymeric films with simple or hybridized TiO_2_ nanoparticles can find applications as catalytic materials for the degradation of hydroxybenzene derivatives under UV or visible light, with several cycles of utilization.

**Figure 12:**
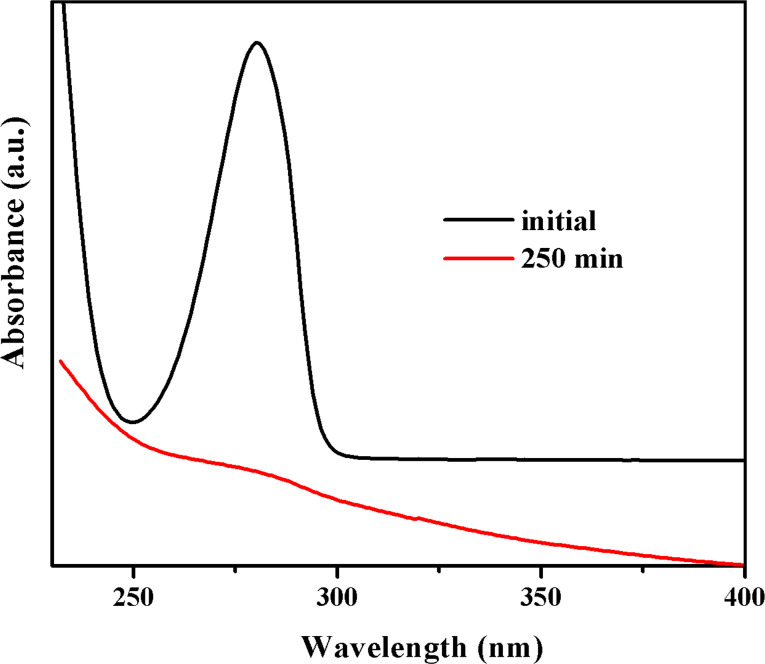
UV–vis absorption spectra of an aqueous dopamine solution before and after 250 min of visible-light irradiation in the presence of TiO_2_/SiO_2_/Fe_2_O_3_ NPs.

For comparison, we also examined the dopamine degradation under visible-light irradiation in presence of TiO_2_/Fe_2_O_3_ or TiO_2_/SiO_2_/Fe_2_O_3_ NPs, and the photolysis degrees attained were 91.2% for TiO_2_/Fe_2_O_3_ NPs and 98.4% for TiO_2_/SiO_2_/Fe_2_O_3_ NPs (Figure 12). The results suggest that the obtained values are slightly higher (by about 3%) than those obtained when hybrid polymeric films were employed as catalysts. A problem raised by experiments with NPs is the recycling of the nanoparticles from the aqueous solution. For instance, the hybridized TiO_2_ NPs can be recovered only after their sedimentation, but even then not entirely. As a result, the employment of the nanoparticles into real aquatic environments can lead to the runoff phenomenon that induces risks to human health and the environment. Given this observation and the fact that the catalytic activity of the hybrid polymeric films is almost the same as that of the inorganic nanoparticles taken alone, as well as the general need of exploitation of the catalysts in several cycles, our composites with simple or hybridized TiO_2_ NPs seem to be a more suitable solution for water treatment under sunlight.

The catalytic performance of the synthesized hybrid polymeric films in the degradation of other pollutants such as dyes (methylene blue, methyl orange, nile red) in a fashion similar to the above molecules should be tested.

## Conclusion

Polymeric composites containing TiO_2_ NPs, pure or combined with Si–O–Si linkages and/or Fe_2_O_3_ NPs were prepared via photopolymerization using oligomeric dimethacrylate and premade NPs. Analysis with XRD, TEM, FTIR and EDX demonstrated the formation of a nanocrystalline anatase phase of TiO_2_ and the uniform distribution of inorganic NPs in the polymer matrix. The hybrid films presented an appropriate photocatalytic activity under UV light in the degradation of phenol derivatives. The decomposition efficiency increased for the composites with TiO_2_ in mixture with other inorganic components. Only the films with TiO_2_ and maghemite nanoparticles exhibited photocatalytic activity under visible irradiation against model pollutants. The presence of Fe_2_O_3_ NPs extended the light absorption of TiO_2_ into the visible range, a feature that recommends their use in water purification under sunlight.

## Experimental

### Materials

Titanium(IV) isopropoxide (TTIP), glacial acetic acid, tetraethyl orthosilicate (TEOS), iron(II) chloride tetrahydrate, iron(III) chloride hexahydrate, absolute alcohol, ammonium hydroxide solution, poly(tetrahydrofuran) (PTHF, *M*_n_ ≈ 2000 g/mol), 2-isocyanatoethyl methacrylate, tetrahydrofuran anhydrous (THF), dibutyltin dilaurate and Irgacure 819 were purchased from Sigma Aldrich Chemical Co. and used without further purification.

### Nanoparticles preparation

#### Synthesis of nanocrystalline TiO_2_

For the preparation of TiO_2_ nanoparticles through the sol–gel method, a literature pathway was employed [49], keeping the molar ratio of TTIP, glacial acetic acid and water at 1:10:350. Thus, 9.3 mL (31.5 mmol) TTIP were hydrolyzed using 18 mL (315 mmol) acetic acid at 0–5 °C. Further, 198 mL water was dropwise added under vigorous stirring for 1 h. The mixture was ultrasonicated for 30 min, and then stirred for another 5 h at room temperature until a clear solution of nanosized TiO_2_ particles was achieved. For the gelation process, the resulting solution was placed into an oven at 70 °C for 12 h, followed by the gel drying at 100 °C. The finely milled sample was calcined at 500 °C for 5 h in the furnace in air to remove the organic substances.

#### Synthesis of TiO_2_ nanoparticles linked with Si–O–Si sequences (TiO_2_/SiO_2_)

The synthesis route followed for the preparation of the second type of nanoparticles is similar to the previously described procedure, excepting that initially, besides 9.3 mL of TTIP 3.07 mL (13.5 mmol) TEOS were added in the reaction vessel (molar ratio of 2.33:1).

#### Synthesis of TiO_2_ nanoparticles combined with maghemite nanoparticles (TiO_2_/Fe_2_O_3_)

The maghemite (γ-Fe_2_O_3_) nanoparticles were synthesized according to a previously published protocol [58]. Thus, 0.025 g Fe_2_O_3_ nanoparticles, calculated on the assumption that the amount of maghemite NPs to be of 1 wt % relatively to the resulting TiO_2_ particles, were added into the reaction vessel, the reaction steps being similar to those followed in the preparation of TiO_2_ NPs.

#### Synthesis of TiO_2_ nanoparticles linked with Si–O–Si and combined with maghemite (TiO_2_/SiO_2_/Fe_2_O_3_)

For the synthesis of TiO_2_/SiO_2_/Fe_2_O_3_ particles, the 1:10:350 ratio of TTIP, glacial acetic acid and water, along with the 2.33:1 molar ratio of TTIP:TEOS were kept, and 1 wt % Fe_2_O_3_ nanoparticles were added. The resulted gel was dried at 100 °C and the finely crushed sample was calcined at 500 °C for 5 h.

#### Synthesis of photopolymerizable urethane dimethacrylate oligomer PTHF-UDMA

**Figure 13:**
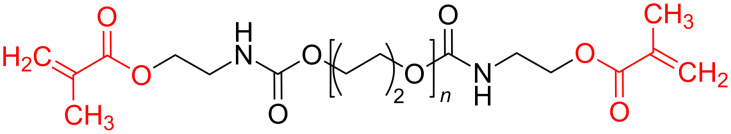
Structure of the photopolymerizable urethane dimethacrylate (PTHF-UDMA).

For the preparation of PTHF-UDMA, 10 g (5 mmol) PTHF were degassed under vacuum for 2 h. Afterwards, 1.44 mL (10 mmol) 2-isocyanatoethyl methacrylate dissolved in 20 ml THF was dropwise added and the mixture was stirred at 40 °C for 12 h in the presence of a catalytic amount of dibutyltin dilaurate. The reaction progress was verified through FTIR spectroscopy following the absorption of the isocyanate stretching band at 2260 cm^−1^ until its disappearance from the spectrum. After the evaporation of the solvent, the urethane dimethacrylate PTHF-UDMA (Figure 13) was collected as colourless viscous liquid (η = 4.46 Pa·s).

**PTHF-UDMA**. Yield: 10.8 g (93.5 %); ^1^H NMR (CDCl_3_, δ) 8.09 (4H, N*H*); 6.06 (d, 2H, C*H*_2_=C in trans position relative to CH_3_ unit); 5.53 (s, 2H, C*H*_2_=C in cis position relative to CH_3_ group); 4.15 (m, 4H, NH-COO-C*H*_2_-CH_2_); 4.01 (m, 4H, COO-C*H*_2_-CH_2_-O); 3.67 (m, 106H, C*H*_2_-CH_2_-CH_2_-C*H*_2_ from PTHF); 3.41 (m, 4H, C*H*_2_-NH-COO); 1.88 (s, 6H, C*H*_3_ linked to double bond); 1.78 (m, 110H, CH_2_-C*H*_2_-C*H*_2_-CH_2_ from PTHF); FTIR (KBr, cm^−1^): 3336 (NH), 2796–2941 (C–H), 1722 (C=O), 1638 (CH_2_=C), 1535 (amide II); 1246, 1113 (C–O–C), 815 (CH_2_=C).

### Preparation of hybrid composites

The hybrid composites were obtained through photopolymerization, as follows: The mixture of PTHF-UDMA monomer (its viscosity permits the attaining of a good dispersion of the inorganic nanoparticles inside the organic matrix), 10 wt % nanoparticles prepared before and small amounts of chloroform was first well mixed in a water bath sonicator for 10 min (in order to ensure a very homogeneous composition of the formulations). Then, these formulations were cast in thin layers on a teflon plate and further subjected to UV irradiation for a period of 600 s, using Irgacure 819 (1 wt %) as photoinitiator, to finally yiled thin films (thickness around 0.2 mm). The UV irradiation was delivered from a Hg–Xe lamp (Hamamatsu Lightningcure Type LC8, Model L9588) with a light intensity of 30 mW·cm^−2^. The photopolymerized films prepared are: S1 – with 10 wt % TiO_2_, S2 – with 10 wt % TiO_2_/SiO_2_, S3 – with 10 wt % TiO_2_/Fe_2_O_3_, and S4 – with 10 wt % TiO_2_/ SiO_2_/Fe_2_O_3_.

### Characterization

The structure of PTHF-UDMA monomer was confirmed by ^1^H NMR spectroscopy using a Bruker Avance DRX 400 spectrometer. The viscosity of PTHF-UDMA monomer was measured with a rotational viscometer RM 100 Touch (Lamy Rheology Instruments, France) using a cone/plate set up (2° cone angle, 40 mm diameter) at varying shear rates of 10–100 s^−1^. The viscosity measurements were performed at 25.0 ± 0.2 °C in triplicate, and the sample volume was kept constant to 0.6 mL. Fourier transform infrared (FTIR) spectra were recorded on a Bruker Vertex 70 FTIR spectrometer. The X-ray diffraction analysis was performed by wide angle X-ray diffraction (WXRD) using a Bruker D8 ADVANCE with Cu Kα radiation (λ = 0.15406 nm), running at an operating voltage of 40 kV and a current of 30 mA. All diffractograms were recorded from 4 to 90° at room temperature with a scan rate of 0.02 °/s. The average crystallite sizes of nanoparticles were calculated by applying the Scherrer equation:


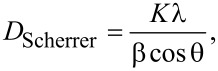


where *D*_Scherrer_ is the average crystallite size, λ is the wavelength of the X-ray radiation (λ = 0.154056 nm for Cu Kα radiation), *K* is the Scherrer constant (*K* = 0.89), β is the full width at half-maximum (FWHM) of the diffraction peak, and θ is the diffraction angle.

Transmission electron microscopy (TEM) analyses were carried out using a HITACHI T7700 microscope operated at 120 kV in high-resolution mode. For these measurements, the nanoparticles were deposited onto a copper grid from a sonicated ethanol solution, while for the hybrid films, the formulations (prepared in the same manner as for the formation of hybrid films) were deposited onto the copper grid and photopolymerized under UV irradiation. The hybrids films formed on the copper grids were investigated by TEM analysis. The micro-chemical analysis of photopolymerized samples was performed using an environmental scanning electron microscope QUANTA200 coupled with an energy dispersive X-ray spectroscope (ESEM/EDX). The film cross-section was examined in low-vacuum mode operating at 20 kV using an LFD detector. The UV–vis absorption spectra of the hybrid polymeric films were measured using a Perkin Elmer Lambda 2 UV–vis spectrophotometer in the wavelength region of 200–700 nm. The total organic carbon (TOC) of the phenol solution before and after visible irradiation in presence of S4 film was measured using a Multi N/C 3100 Analyticjena analyzer.

### Photocatalytic activity measurements

In a manner analogous to [59], the photocatalytic activity of the synthesized hybrid films was evaluated following the degradation of phenol, hydroquinone or dopamine in aqueous solutions under ambient conditions and UV–visible irradiation. A piece of each composite film (1 g) was added into 50 mL of phenol (1.06 × 10^−3^ M) or hydroquinone/dopamine (0.5 × 10^−3^ M) aqueous solutions. The mixture was then constantly irradiated with UV light (Hg–Xe lamp, λ = 365 nm, light intensity ca. 8 mW·cm^−2^) or visible light source (Xe lamp, λ = 400–800 nm). 1.5 mL of the irradiated solution was collected at given time intervals and further analysed using an UV–vis spectrophotometer (Perkin Elmer Lambda 2). For the parallel experiments: 0.1 g of TiO_2_/Fe_2_O_3_ NPs or TiO2/SiO_2_/Fe_2_O_3_ NPs was added in 50 mL dopamine (0.5 × 10^−3^ M) aqueous solutions and the mixture was kept under vigorous stirring and constantly irradiated with visible light. The pollutants solutions without hybrid films or with simple polymeric film (without TiO_2_ NPs) were irradiated for 250 min. Also, the S1 and S4 films were added in the pollutants solutions and kept in the dark for the same period of time, and finally, all solutions were evaluated by UV–vis and fluorescence (Perkin-Elmer LS 55) spectroscopy (in the case of dopamine solution). The extinction coefficients of phenol, hydroquinone and dopamine were independently measured to quantify the evolution of the concentration of the pollutants.

## References

[1] O’Regan B, Grätzel M (1991). Nature.

[2] Adachi M, Murata Y, Takao J, Jiu J, Sakamoto M, Wang F (2004). J Am Chem Soc.

[3] Subramanian V, Karki A, Gnanasekar K I, Eddy F P, Rambabu B J (2006). J Power Sources.

[4] Zhu Y F, Shi J J, Zhang Z Y, Zhang C, Zhang X R (2002). Anal Chem.

[5] Thurn K T, Paunesku T, Wu A G, Brown E M B, Lai B, Vogt S, Maser J, Aslam M, Dravid V, Bergan R (2009). Small.

[6] Zeng L Y, Ren W Z, Xiang L C, Zheng J J, Chen B, Wu A G (2013). Nanoscale.

[7] Ren W Z, Zeng L Y, Shen Z Y, Xiang L C, Gong A, Zhang J C, Mao C G, Li A G, Paunesku T, Woloschak G E (2013). RSC Adv.

[8] Fujishima A, Honda K (1972). Nature.

[9] Han L, Wang P, Zhu C, Zhai Y, Dong S (2011). Nanoscale.

[10] Horiguchi Y, Kanda T, Torigoe K, Sakai H, Abe M (2014). Langmuir.

[11] Khataee A R, Kasiri M B (2010). J Mol Catal A: Chem.

[12] Nakata K, Fujishima A (2012). J Photochem Photobiol, C.

[13] He Y, Zhang L, Teng B, Fan M (2015). Environ Sci Technol.

[14] Wang Y, Lai Q, Zhang F, Shen X, Fan M, He Y, Ren S (2014). RSC Adv.

[15] He Y, Wang Y, Zhang L, Teng B, Fan M (2015). Appl Catal, B.

[16] Xu Y-J, Zhuang Y, Fu X (2010). J Phys Chem C.

[17] Wang D-H, Jia L, Wu X-L, Lu L-Q, Xu A-W (2012). Nanoscale.

[18] Dunnill C W, Parkin I P (2011). Dalton Trans.

[19] Luo W Q, Fu C Y, Li R F, Liu Y S, Zhu H M, Chen X Y (2011). Small.

[20] Hafez H, Saif M, Abdel-Mottaleb M S A (2011). J Power Sources.

[21] Zhu J F, Deng Z G, Chen F, Zhang J L, Chen H J, Anpo M, Huang J Z, Zhang L Z (2006). Appl Catal, B.

[22] Cha W, Le H A, Chin S, Kim M, Jung H, Yun S-T, Jurng J (2013). Mater Res Bull.

[23] Janisch R, Gopal P, Spaldin N A (2005). J Phys: Condens Matter.

[24] Li Y Z, Jin S F, Xie H, Chen X, Tian T T, Zhao X J (2012). J Mater Chem.

[25] Chibac A L, Melinte V, Buruiana T, Mangalagiu I, Buruiana E C (2015). J Polym Sci, Part A: Polym Chem.

[26] Liu L C, Gu X R, Sun C Z, Li H, Deng Y, Gao F, Dong L (2012). Nanoscale.

[27] Lezner M, Grabowska E, Zaleska A (2012). Physicochem Probl Miner Process.

[28] Niu H L, Wang Q M, Liang H X, Chen M, Mao C J, Song J M, Zhang S Y, Gao Y H, Chen C L (2014). Materials.

[29] Su J W, Zhang Y X, Xu S C, Wang S, Ding H L, Pan S S, Wang G Z, Li G H, Zhao H J (2014). Nanoscale.

[30] Yao H B, Fan M H, Wang Y J, Luo G S, Fei W Y (2015). J Mater Chem A.

[31] Arabatzis I M, Antonaraki S, Stergiopoulos T, Hiskia A, Papaconstantinou E, Bernard M C, Falaras P (2002). J Photochem Photobiol, A.

[32] Shan A Y, Ghazi T I M, Rashid S A (2010). Appl Catal, A.

[33] Gao Y, Chen B H, Li H L, Ma Y X (2003). Mater Chem Phys.

[34] Chen F H, Yan F F, Chen Q T, Wang Y W, Han L F, Chen Z J, Fang S M (2014). Dalton Trans.

[35] Mukherjee D, Barghi S, Ray A K (2014). Processes.

[36] Jallouli N, Elghniji K, Trabelsi H, Ksibi M (2014). Arabian J Chem.

[37] Lei P, Wang F, Gao X, Ding Y, Zhang S, Zhao J, Liu S, Yang M (2012). J Hazard Mater.

[38] Liu Y, Wei J H, Xiong R, Pan C X, Shi J (2011). Appl Surf Sci.

[39] Janáky C, de Tacconi N R, Chanmanee W, Rajeshwar K (2012). J Phys Chem C.

[40] Li X C, Sun J S, He G H, Jiang G L, Tan Y, Xue B (2013). J Colloid Interface Sci.

[41] Ullah H, Tahir A A, Mallick T K (2016). Sens Actuators, B.

[42] Stewart B D, Andrews L G, Pelletier B S, Daly C A, Boyd J E (2015). J Water Process Eng.

[43] Yin J, Deng B (2015). J Membr Sci.

[44] Man Y, Mu L, Wang Y, Lin S, Rempel G L, Pan Q (2015). Polym Compos.

[45] Yagci Y, Jockusch S, Turro N J (2010). Macromolecules.

[46] Chatani S, Kloxin C J, Bowman C N (2014). Polym Chem.

[47] Deng F, Li Y, Luo X, Yang L, Tu X (2012). Colloids Surf, A.

[48] Balachandran K, Venckatesh R, Sivaraj R, Rajiv P (2014). Spectrochim Acta, Part A.

[49] Venkatachalam N, Palanichamy M, Murugesan V (2007). Mater Chem Phys.

[50] Shen Z-Y, Li L-Y, Li Y, Wang C-C (2011). J Colloid Interface Sci.

[51] Song C F, Lü M K, Yang P, Xu D, Yuan D R (2002). Thin Solid Films.

[52] Zhao X, Lv L, Pan B C, Zhang W M, Zhang S J, Zhang Q X (2011). Chem Eng J.

[53] Fujishima A, Zhang X T, Tryk D A (2008). Surf Sci Rep.

[54] Xiang Q J, Yu J G, Jaroniec M (2011). Chem Commun.

[55] Yu J-G, Yu H-G, Cheng B, Zhao X-J, Yu J C, Ho W-K (2003). J Phys Chem B.

[56] Ye M M, Zhang Q, Hu Y X, Ge J P, Lu Z D, He L, Chen Z L, Yin Y D (2010). Chem – Eur J.

[57] Liu Z Y, Quan X, Fu H B, Li X Y, Yang K (2004). Appl Catal, B.

[58] Wu Z-G, Wang Y (2013). Mater Sci-Pol.

[59] Chibac A L, Buruiana T, Melinte V, Mangalagiu I, Buruiana E C (2015). RSC Adv.

